# Reactive oxygen species formation and bystander effects in gradient irradiation on human breast cancer cells

**DOI:** 10.18632/oncotarget.9517

**Published:** 2016-05-20

**Authors:** Dongqing Zhang, Tingyang Zhou, Feng He, Yi Rong, Shin Hee Lee, Shiyong Wu, Li Zuo

**Affiliations:** ^1^ Radiologic Sciences and Respiratory Therapy Division, School of Health and Rehabilitation Sciences, The Ohio State University College of Medicine, Columbus, OH 43210, USA; ^2^ Department of Radiation Oncology, The James Cancer Hospital, The Ohio State University Wexner Medical Center, Columbus, OH 43210, USA; ^3^ Department of Health and Exercise Sciences, Skidmore College, Saratoga Springs, NY 12866, USA; ^4^ Davis Heart and Lung Research Institute, The Ohio State University Wexner Medical Center, Columbus, OH 43210, USA; ^5^ Interdisciplinary Biophysics Graduate Program, The Ohio State University, Columbus, OH 43210, USA; ^6^ Urology Nevada/Northern Nevada Radiation Oncology, Reno, NV 89521, USA; ^7^ Edison Biotechnology Institute, Ohio University, Athens, OH 45701, USA; ^8^ Department of Chemistry and Biochemistry, Molecular and Cellular Biology Program, Ohio University, Athens, OH 45701, USA

**Keywords:** bystander effects, reactive oxygen species, gradient irradiation, breast cancer cells, MCF-7

## Abstract

Ionizing radiation (IR) in cancer radiotherapy can induce damage to neighboring cells via non-targeted effects by irradiated cells. These so-called bystander effects remain an area of interest as it may provide enhanced efficacy in killing carcinomas with minimal radiation. It is well known that reactive oxygen species (ROS) are ubiquitous among most biological activities. However, the role of ROS in bystander effects has not been thoroughly elucidated. We hypothesized that gradient irradiation (GI) has enhanced therapeutic effects via the ROS-mediated bystander pathways as compared to uniform irradiation (UI). We evaluated ROS generation, viability, and apoptosis in breast cancer cells (MCF-7) exposed to UI (5 Gy) or GI (8–2 Gy) in radiation fields at 2, 24 and 48 h after IR. We found that extracellular ROS release induced by GI was higher than that by UI at both 24 h (*p* < 0.001) and 48 h (*p* < 0.001). More apoptosis and less viability were observed in GI when compared to UI at either 24 h or 48 h after irradiation. The mean effective doses (ED) of GI were ~130% (24 h) and ~48% (48 h) higher than that of UI, respectively. Our results suggest that GI is superior to UI regarding redox mechanisms, ED, and toxic dosage to surrounding tissues.

## INTRODUCTION

Previous research has shown that ionizing radiation (IR) can induce cells to emit signals that affect neighboring cells, termed non-targeted effects (NTE) [1, 2]. These studies show that tissues or organs respond collectively to IR dose damages which include both direct effects and NTE. One significant class of NTE, the bystander effects, is typically observed in less- or un-irradiated cell. Different bystander signaling-mediated mechanisms have been proposed (e.g., through growth medium, inflammatory cytokines, reactive oxygen species (ROS), etc.) [3]. Cells that are exposed to bystander signals experience adverse effects including cell destruction, DNA damage, and gene mutation [4]. Despite some promising investigations on the radiation bystander effects, gaps still exist in the understanding of the quantitative aspects and its impact on radiobiological models.

Bystander effects may also play an important role in radiotherapy. For example, optimized non-uniform (e.g., gradient) dose delivery to the tumor target may significantly reduce the radiation toxicity to healthy cells near the target. Cell survival can be enhanced by the less damage to neighboring cells subjected to different types of intensity-modulated radiation fields compared with uniform irradiation (UI) [5]. Tumor cells' response to non-uniform irradiation is more sensitive than traditional UI due to a different cellular communication mechanism [6]. Buonanno et al. suggested that bystander effects were significantly dependent on IR quality and dosage [7]. Progress in understanding bystander effects can improve radiobiological modeling of tumor and normal tissues under various IR dosing schemes. Bystander effects resulting from exposures to low to high Linear-Energy-Transfer (LET) radiation have been studied in the past decades [7–11]. Modern cancer radiotherapy, such as intensity modulated radiotherapy (IMRT) [12, 13], uses multileaf collimator (MLC) to create non-uniform modulated radiation fields with shapes conformed to the tumor contour while sparing the surrounding healthy tissues or organs [14]. However, models of cell dosage response to the non-uniform modulated radiation fields are not well established and the NTE (bystander effects) are ignored. The commonly used Linear-Quadratic model is merely used to estimate the biologically effective doses (ED) for UI fields [15]. Accordingly, new radiobiological models should take into account the non-uniform radiation fields and the NTE (bystander effects).

Since ROS are ubiquitous among biological activities [16], we seek to understand how the bystander effects are mediated via redox mechanisms. Chen et al.'s *in vitro* study (e.g., human lung cancer cells) confirmed that ROS play some roles in the bystander effects induced by low-dose-rate seed irradiation [17]. Interestingly, bystander cells co-cultured with irradiated cells persistently exhibit marked levels of oxidative stress [7]. Further studies have shown that the cellular signaling cascade of bystander response may involve mediators such as interleukin (IL)-6, IL-8, tumor necrosis factor-alpha (TNF-α), ROS, and reactive nitrogen species [3]. As ROS levels increase in response to irradiation-induced bystander effects [18], evaluation of ROS-oriented pathways induced by IR become necessary in order to understand the complex bystander mechanism. We hypothesized that by spreading the bystander signals to less-irradiated regions, gradient dose delivery is able to achieve this beneficial effect without sacrificing the efficacy of killing malignant tumor cells. Accordingly, we investigated the potential bystander effects of gradient irradiation (GI) on human breast cancer cells (MCF-7) by exploring the distinct molecular redox interactions between ROS and antioxidants. This approach will expand our knowledge of the bystander effects induced by different radiation strategies and the underlying redox mechanism, shedding light on future breast cancer radiotherapy.

## RESULTS

### Extracellular ROS formation was higher in GI (8–2 Gy) than UI (5 Gy)

**Figure 1:**
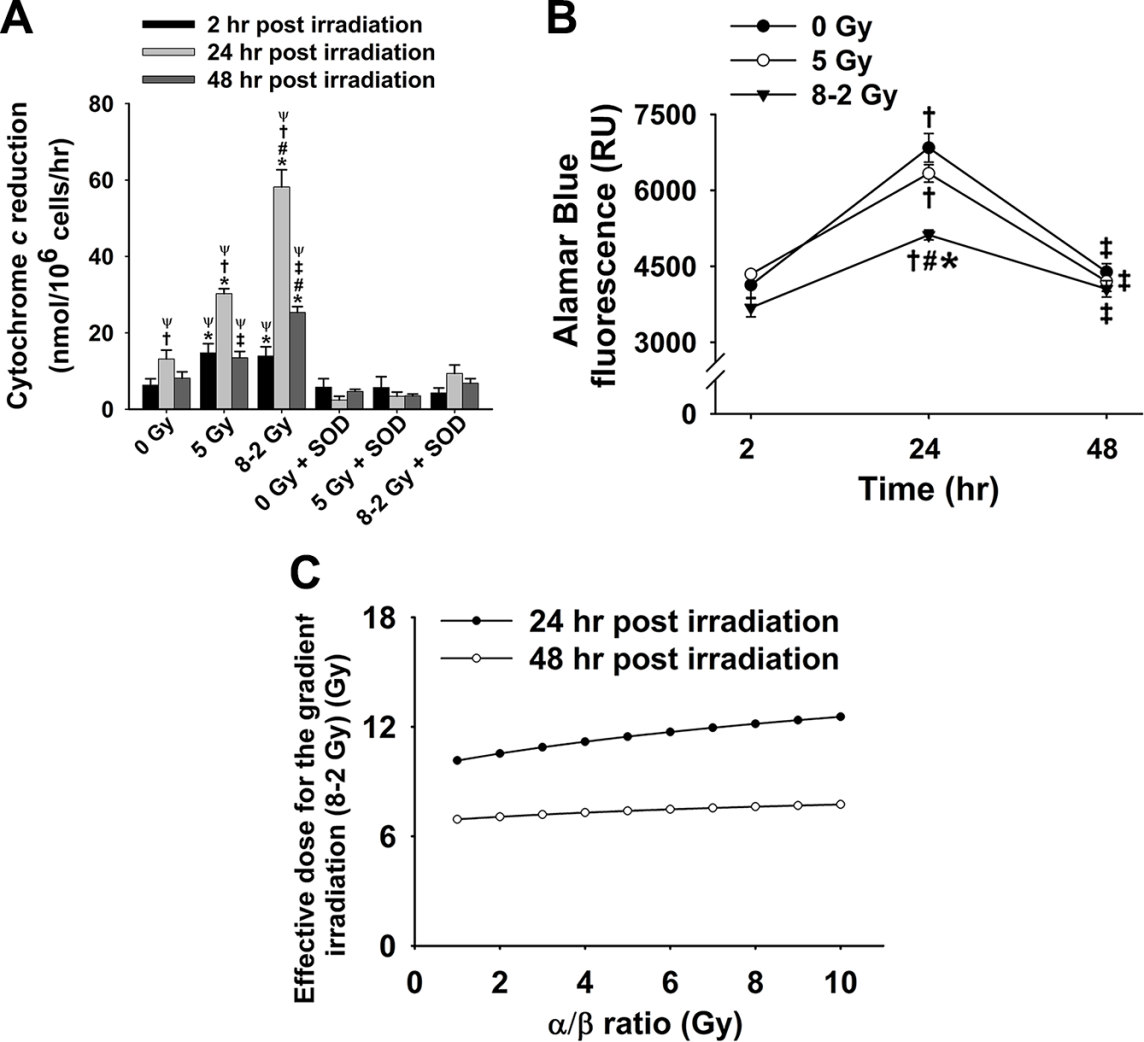
Cytochrome *c* reduction as well as corresponding cell viability and ED after irradiation (mean ± SE) (**A**) Rate of cytochrome *c* reduction at 2, 24 and 48 h (*n* = 6); 0 Gy ± SOD *vs*. 5 Gy ± SOD, and 8–2 Gy ± SOD; (**B**) Cell viability represented by Alamar Blue fluorescence at 2, 24 and 48 h (*n* = 3); (**C**) Estimated ED for GI (8–2 Gy) by comparing the fluorescence data to that of UI (5 Gy), with α/β ratio from 1–10 Gy at 24 h and 48 h. ^*^p < 0.05 *vs*. 0 Gy at the same time; ^#^*p* < 0.05 *vs*. 5 Gy at the same time; ^Ψ^*p* < 0.05 *vs*. SOD groups at the same time and at the same dose; ^†^*p* < 0.05 *vs*. 2 h point at the same dose; ^‡^*p* < 0.05 *vs*. 24 h point at the same dose.

Extracellular ROS formation was monitored and represented by cytochrome *c* reduction as shown in Figure 1A. Both UI (5 Gy) and GI (8–2 Gy) significantly stimulated ROS release when compared to the control group (0 Gy) at 2 h (*p* < 0.05 for both UI and GI), 24 h (*p* < 0.005 for both UI and GI), and 48 h (*p* < 0.001 for GI). Superoxide dismutase (SOD, 1500 U/mL), a membrane-impermeable scavenger of extracellular superoxide (O_2_^·−^), effectively diminished this irradiation-induced ROS elevation. Furthermore, extracellular O_2_^·−^ levels demonstrate a significant increase from 2 h to 24 h after irradiation, followed by a decline at 48 h for all three treatment groups (control, UI and GI). By comparing the UI and GI groups, we found that GI stimulated more ROS release as compared to UI at both 24 h (*p* < 0.001) and 48 h (*p* < 0.001), indicating a stronger redox-mediated bystander signal in the medium under GI.

### Cell viability was more reduced by GI (8–2 Gy) than UI (5 Gy)

The average cell viability in each dish was evaluated at 2 h, 24 h, and 48 h after irradiation by Alamar Blue and Trypan Blue. As shown in Figure 1B, the results (Alamar Blue) indicate that MCF-7 cells proliferated markedly from 2 h to 24 h in all three treatments, followed by a significant decrease in cell viability at 48 h after irradiation. Notably, GI (8–2 Gy)-treated cells displayed the lowest cell viability as compared to the control (0 Gy) (*p* < 0.005) and UI (5 Gy) groups (*p* < 0.05) at 24 h. However, at 48h after irradiation, the three treatment groups demonstrate no significant difference in terms of the cell viability. Moreover, based on the cell viability data, the ED for GI (8–2 Gy) was estimated to quantitatively demonstrate more superior therapeutic effect of GI, which is likely attributed to the presence of bystander effects. The cell dose-survival curve is described by linear-quadratic models as the equation below [15, 19],

$$S\;=\;\exp(-\alpha D-\beta D^{2})$$

where *S* is the cell survival fraction with irradiated dose *D*, and α and β are coefficients for the linear and quadratic dose terms. The α/β ratio (in dose unit of Gy) represents an important characteristic dose point, at which the cell killing effects from the linear and quadratic terms are equal. The quantification of α/β is used to describe the tissue response to dose fractionation, in order to determine the correct dose regimens [20]. In this study, the measured Alamar Blue fluorescence (Figure 1B) was regarded as positively correlated to cell survival fraction after irradiation [21]. Assuming the measured fluorescence is proportional to the cell surviving fraction [21], the mean cell survival fractions can be estimated by normalizing to the control group (0 Gy): at 24 h after irradiation, the survival fractions were 92.6% and 74.8% for the UI (5 Gy) and GI (8–2 Gy), respectively; at 48 h after irradiation, the mean survival fractions were 95.8% and 92.4% for the UI (5 Gy) and GI (8–2 Gy), respectively. Then based on the linear-quadratic model, the ED associated with GI can be estimated as a function of the α/β ratio by comparing to the survival fractions associated with UI, shown in Figure 1C.

**Figure 2:**
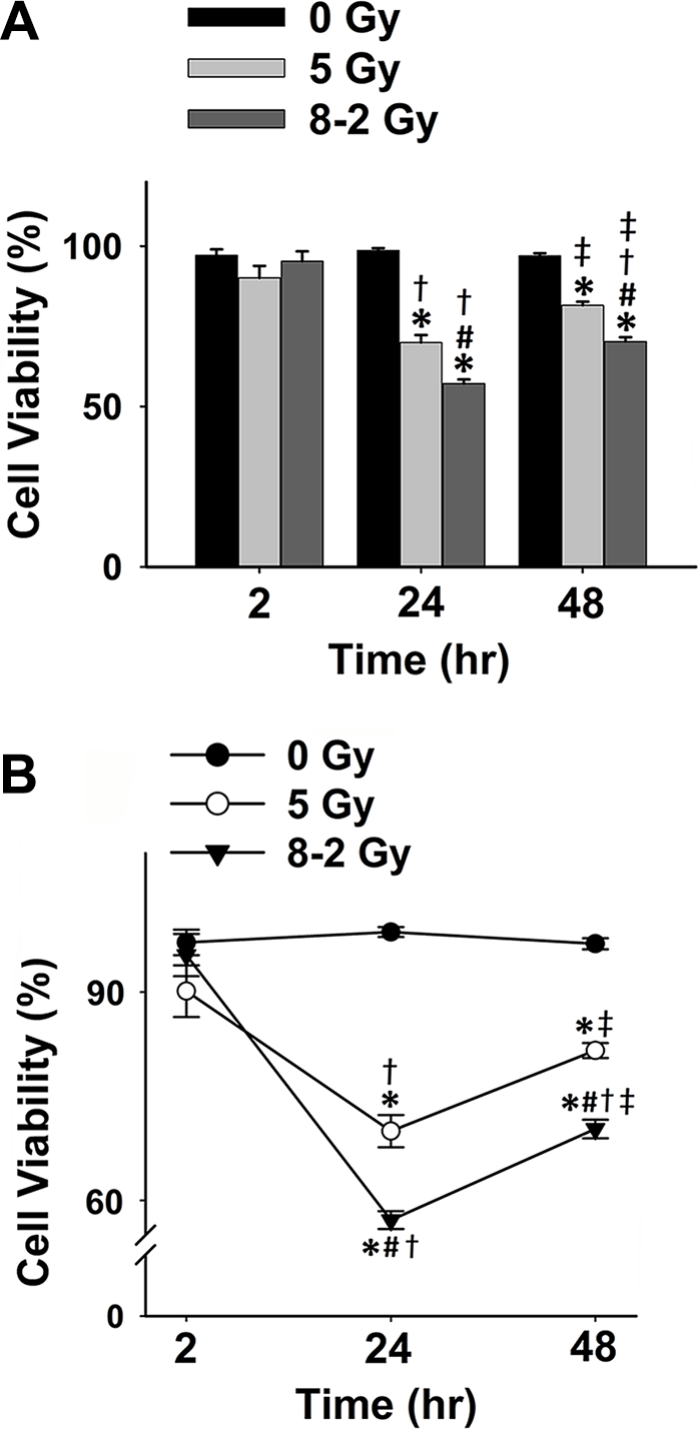
Cell viability measured using Trypan Blue for 0 Gy, 5 Gy, and 8–2 Gy (measured from 6–9 randomly selected areas of ~300–900 cells on the Trypan Blue assay slides, respectively; mean ± SE) (**A**) Cell viability represented by a bar chart at 2, 24, and 48 h after irradiation. (**B**) Cell viability represented by a line chart at 2, 24, and 48 h after irradiation. ^*^p < 0.05 *vs*. 0 Gy at the same time; ^#^*p* < 0.05 *vs*. 5 Gy at the same time; ^†^*p* < 0.05 *vs*. 2 h at the same dose; ^‡^*p* < 0.05 *vs*. 24 h at the same dose.

The results from Trypan Blue (cell death marker) assay showed a similar cell viability trend to the data from Alamar Blue assay under both UI and GI (Figure 2). At 2 h after irradiation, no difference in cell viability was observed between the irradiation groups (GI and UI) and control. Both UI (5 Gy) (*p* < 0.001) and GI (8–2 Gy) (*p* < 0.001) groups showed a decreased cell survival at 24 h following irradiation compared to data at 2 h (Figure 2B). At 48 h, irradiated cell viability was increased compared to data at 24 h (UI: *p* < 0.05; GI: *p* < 0.001) which, however, were still largely lower than that of the control at 48 h (*p* < 0.001 for both UI and GI). GI groups (8–2 Gy) continue to display lower cell survival rate than that in UI (5 Gy) groups at both 24 h (*p* < 0.001) and 48 h (*p* < 0.001) after irradiation (Figure 2).

**Figure 3:**
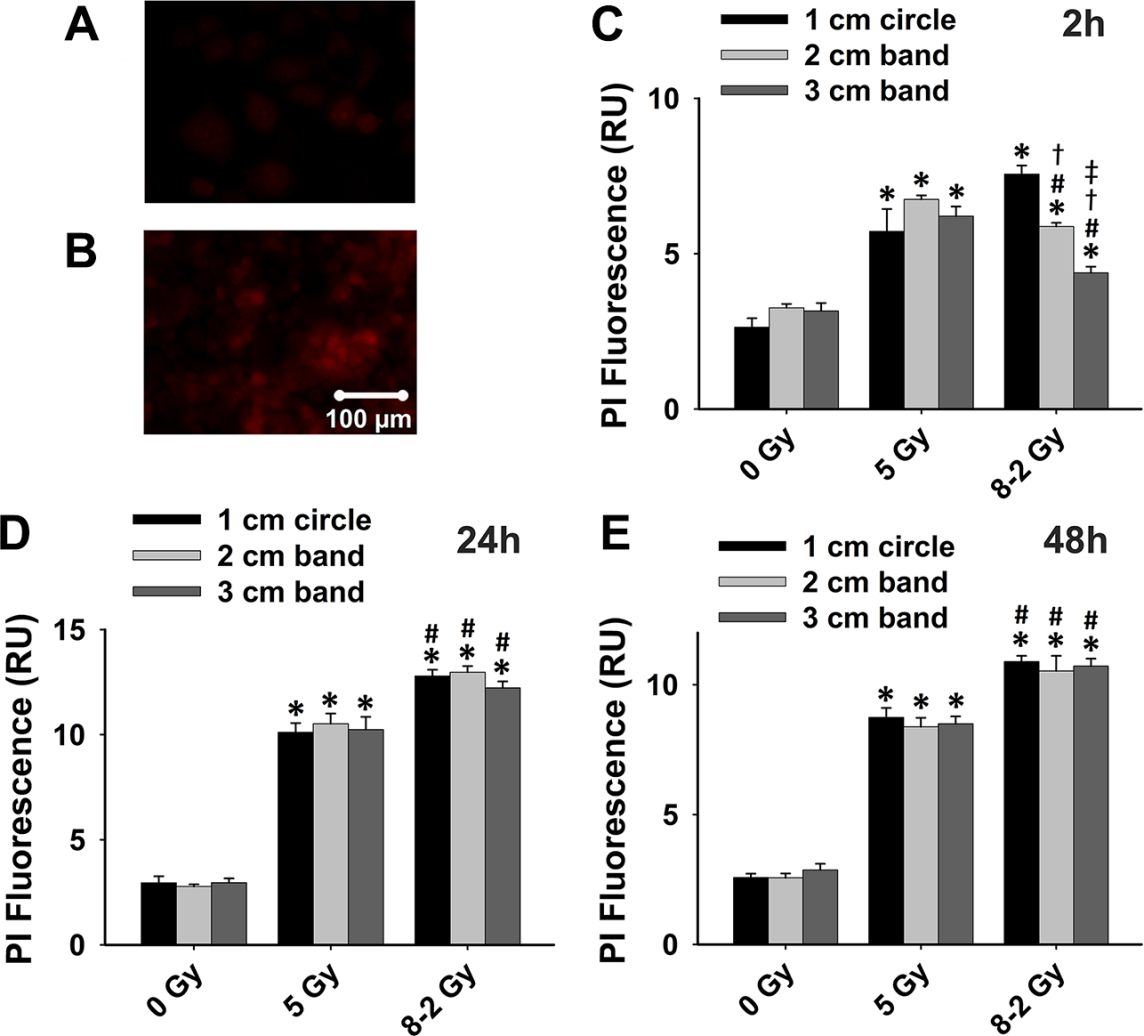
Cell viability detected using PI fluorescence (shown by red fluorescence; measured from three randomly selected areas of 300–500 cells in 1 cm circle, 2 and 3 cm bands, respectively); 0 Gy *vs*. 5 Gy *vs*. 8–2 Gy (**A**) Representative image of high cell viability shown by PI fluorescence. (**B**) Representative image of low cell viability shown by PI fluorescence. (**C**) Grouped PI fluorescence measured from 1 cm circle, 2 and 3 cm bands at 2 h after irradiation. (**D**) Grouped PI fluorescence measured from 1 cm circle, 2 and 3 cm bands at 24 h after irradiation. (**E**) Grouped PI fluorescence measured from 1 cm circle, 2 and 3 cm bands at 48 h after irradiation. Data were presented as mean ± SE; ^*^p < 0.05 *vs*. 0 Gy at the same circle/band; ^#^*p* < 0.05 *vs*. 5 Gy at the same circle/band; ^†^*p* < 0.05 *vs*. 1 cm circle at the same dose; ^‡^*p* < 0.05 *vs*. 2 cm band at the same dose.

**Figure 4:**
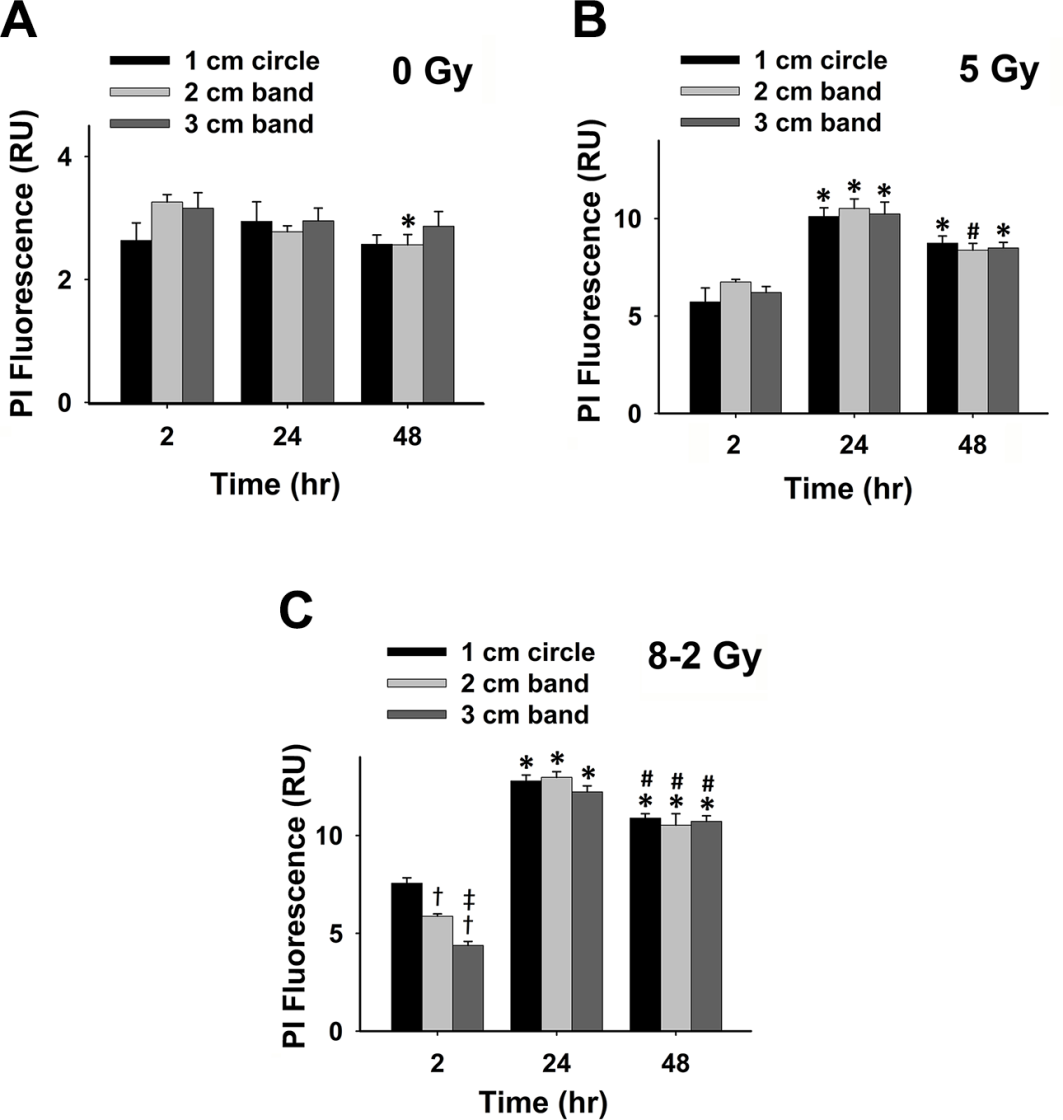
Grouped data of cell viability represented by PI fluorescence; 2 h *vs* 24 h *vs*. 48 h (measured from three randomly selected areas of 300–500 cells in 1 cm circle, 2 and 3 cm bands, respectively; mean ± SE). (**A**) PI fluorescence measured from 1 cm circle, 2 and 3 cm bands of control. (**B**) PI fluorescence measured at 1 cm circle, 2 and 3 cm bands in UI (5 Gy). (**C**) PI fluorescence measured at 1 cm circle, 2 and 3 cm bands in GI (8–2 Gy). ^*^p < 0.05 *vs*. 2 h at the same circle/band; ^#^*p* < 0.05 *vs*. 24 h at the same circle/band; ^†^*p* < 0.05 *vs*. 1 cm circle at the same time; ^‡^*p* < 0.05 *vs*. 2 cm band at the same time.

Propidium iodide (PI), another cell death marker, was used to evaluate cell viability spatially at different regions of the dish (i.e., 1–3 cm bands) under both UI and GI. Based on the manufactory protocol, a higher intensity of PI fluorescence represents a lower cell viability (Figure 3A and 3B). As shown in Figure 3C, 3D, and 3E, irradiated cells (both UI and GI) emitted stronger fluorescence as compared to the control at 2 h, 24 h, and 48 h (*p* < 0.05). At 2 h, the cell viability of GI (8–2 Gy) showed a decreased trend from a 1 cm circle to 3 cm band, which is consistent with the irradiation intensity profile of GI (8–2 Gy). Accordingly, the survival rate in the 3 cm band was significantly higher than that of the 2 cm band in GI (8–2 Gy) groups (*p* < 0.01). Cell viability of both regions (2 and 3 cm) were higher than that of the 1 cm circle (*p* < 0.005) in GI groups (Figure 3C). It is noted that at 2 h, UI (5 Gy) groups displayed lower cell survival rates than that of GI groups (8–2 Gy) in both 2 and 3 cm bands (*p* < 0.01) (Figure 3C). However, at 24 h, the cell viability of GI (8–2 Gy) was decreased to a much lowered level than that of UI (5 Gy) in all three regions (*p* < 0.01 for 1 cm circle, *p* < 0.01 for 2 cm band, and *p* < 0.05 for 3 cm band). In addition, cell viability showed little difference between the three regions of GI (8–2 Gy) or UI (5 Gy) at 24 h (Figure 3D). At 48 h after irradiation, GI (8–2 Gy)-treated cells still displayed lower viability as compared to those under UI (5 Gy) in all three regions (*p* < 0.01 for 1 cm circle, *p* < 0.05 for 2 cm band, and *p* < 0.01 for 3 cm band). No difference of cell survival was observed among three regions of either GI (8–2 Gy) or UI (5 Gy) at 48 h (Figure 3E). Furthermore, consistent with the results from both Alamar Blue and Trypan Blue assay, the control group (0 Gy) demonstrated little changes of cell viability at 2 h, 24 h and 48 h in all three regions (Figure 4A). The cell viability in both GI (8–2 Gy) and UI (5 Gy) decreased at 24 h compared to data at 2 h (*p* < 0.05); however, the cell viability showed a trend of elevation at 48 h in all the three regions compared to that at 24 h (Figure 4B and 4C).

### Cellular apoptosis was more induced in GI (8–2 Gy) than UI (5 Gy)

**Figure 5:**
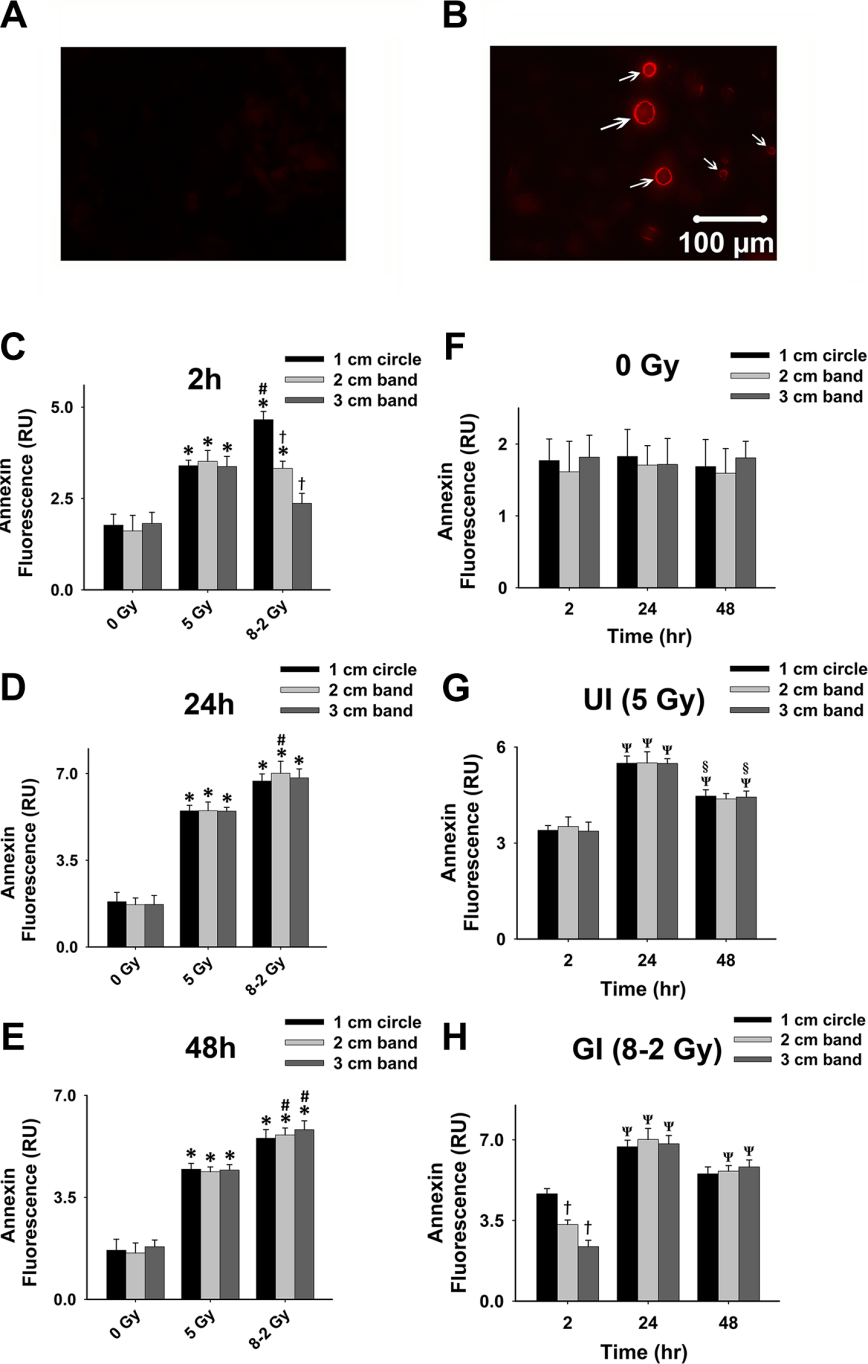
Cell apoptosis at 2, 24, and 48 h after irradiation Cell apoptosis was detected using Annexin V-Cy3 shown by red fluorescence; measured from three randomly selected areas of 300–500 cells in 1 cm circle, 2 and 3 cm bands, respectively. (**A**) Representative image of control cells stained with Annexin V-Cy3. (**B**) Representative image of apoptotic cells stained with Annexin V-Cy3; apoptotic cells emitted red fluorescence on membranes (arrows). (**C**) Grouped Annexin fluorescence in 1 cm circle, 2 and 3 cm bands at 2 h after irradiation. (**D**) Grouped Annexin fluorescence in 1 cm circle, 2 and 3 cm bands at 24 h after irradiation. (**E**) Grouped Annexin fluorescence in 1 cm circle, 2 and 3 cm bands at 48 h after irradiation. (**F**) Grouped Annexin fluorescence in 1 cm circle, 2 and 3 cm bands of control. (**G**) Grouped Annexin fluorescence in 1 cm circle, 2 and 3 cm bands in UI (5 Gy). (**H**) Grouped Annexin fluorescence in 1 cm circle, 2 and 3 cm bands in GI (8–2 Gy). Grouped data were presented as mean ± SE; ^*^p < 0.05 *vs*. 0 Gy at the same irradiation circle/band; ^#^*p* < 0.05 *vs*. 5 Gy at the same irradiation circle/band; ^†^*p* < 0.05 *vs*. 1 cm circle at the same dose. ^Ψ^*p* < 0.05 *vs*. 2 h at the same circle/band; ^§^*p* < 0.05 *vs*. 24 h at the same circle/band.

As represented by Annexin V-Cy3 fluorescence, apoptosis occurred after irradiation in MCF-7 cells (Figure 5). At 2 h, all regions of UI (5 Gy), as well as the 1 cm circle and 2 cm band of GI (8–2 Gy), exhibited higher apoptotic levels than control. At this time point, the apoptotic levels were decreased from 1 cm circle to 2 cm band, as well as from 2 cm to 3 cm bands under GI (8–2 Gy), consistent with the intensity profile of GI (8–2 Gy) (Figure 5C). In addition, apoptosis levels in 1 cm circle regions of GI (8–2 Gy) were higher than those of the 1 cm circle areas of UI (5 Gy) (*p* < 0.05), while no significant difference was observed in 2 and 3 cm bands between GI and UI. At 24 h, GI (8–2 Gy) showed higher apoptotic levels compared to UI (5 Gy) (*p* < 0.05 in 2 cm band), while there was little difference in apoptotic levels among three regions of GI (8–2 Gy) (Figure 5D). At 48 h, Annexin fluorescence signals were stronger in the GI group (8–2 Gy) as compared to that in the UI (5 Gy) group (*p* < 0.05 in 2 and 3 cm bands, Figure 5E). There were no changes of apoptotic conditions in control groups (0 Gy) at 2 h, 24 h, and 48 h (Figure 5F). Both UI (5 Gy)-and GI (8–2 Gy)-treated cells displayed higher apoptosis levels than the control group in all three regions at both 24 and 48 h as shown in Figure 5D and 5E (*p* < 0.01 for UI at both 24 and 48 h; *p* < 0.001 for GI at both 24 and 48 h). Both UI (5 Gy)-and GI (8–2 Gy)-treated cells also showed increased levels of apoptosis at 24 h (*p* < 0.05), followed by a trend of decline at 48 h in all three regions (Figure 5G and 5H).

### Intracellular ROS levels following UI (5 Gy) and GI (8–2 Gy) were elevated in different patterns but ended at similar levels

**Figure 6:**
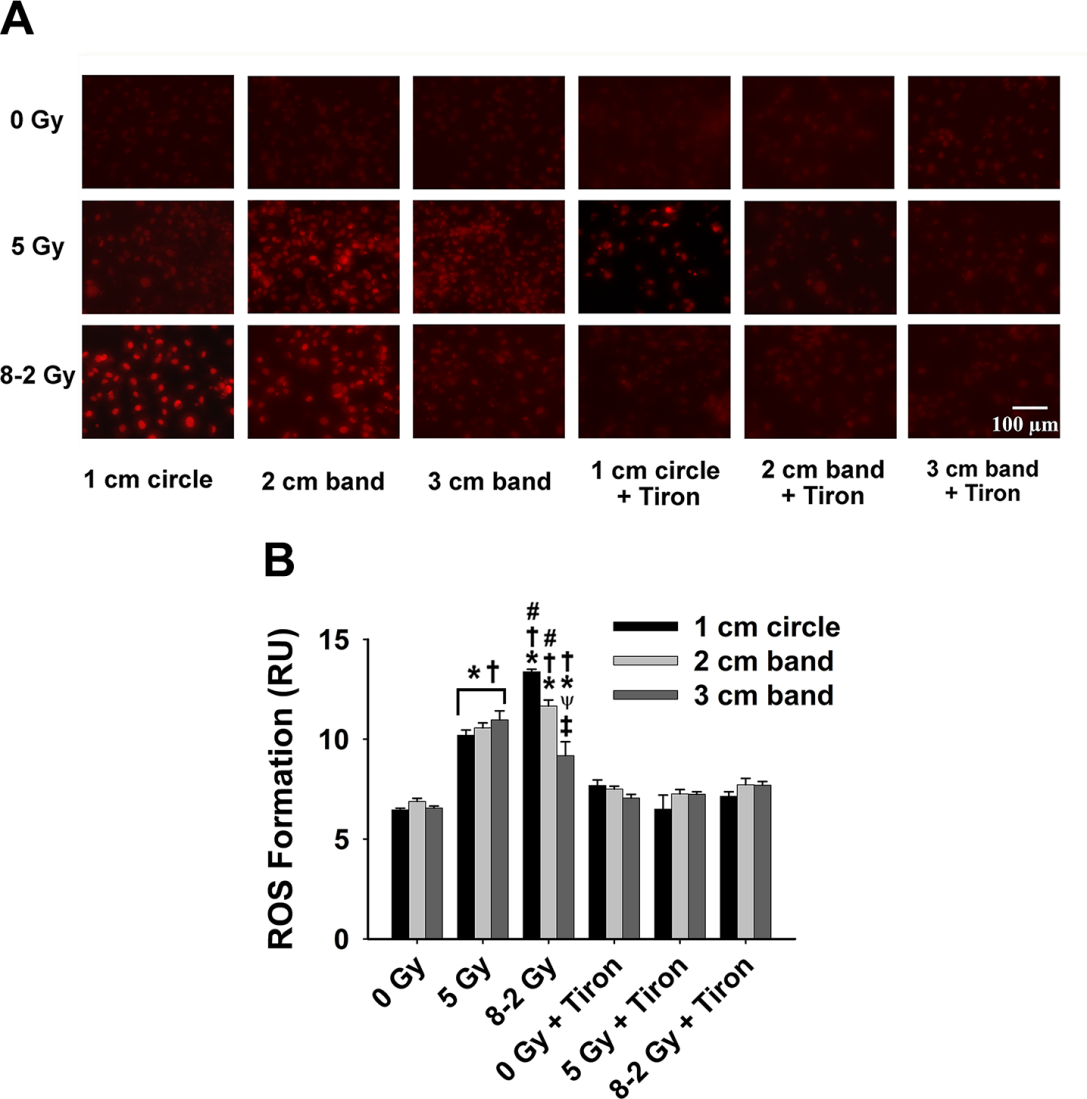
Intracellular ROS formation at 2 h after irradiation detected by DHE/ET fluorescence; data were measured from three randomly selected areas of 300*−*500 cells in 1 cm circle, 2 and 3 cm bands, respectively; 0 Gy ± Tiron *vs*. 5 Gy ± Tiron, and 8–2 Gy ± Tiron (**A**) Representative images of ROS formation (shown by red fluorescence); (**B**) Grouped ROS formation in 1 cm circle, 2 and 3 cm bands (mean ± SE); ^*^p < 0.05 *vs*. 0 Gy at the same circle/band; ^#^*p* < 0.05 *vs*. 5 Gy at the same circle/band; ^†^*p* < 0.05 *vs*. Tiron-treated group at the same circle/band and at the same dose; ^ψ^*p* < 0.05 *vs*. 1 cm circle within the same treatment; ^‡^*p* < 0.05 *vs*. 2 cm band within the same treatment.

**Figure 7:**
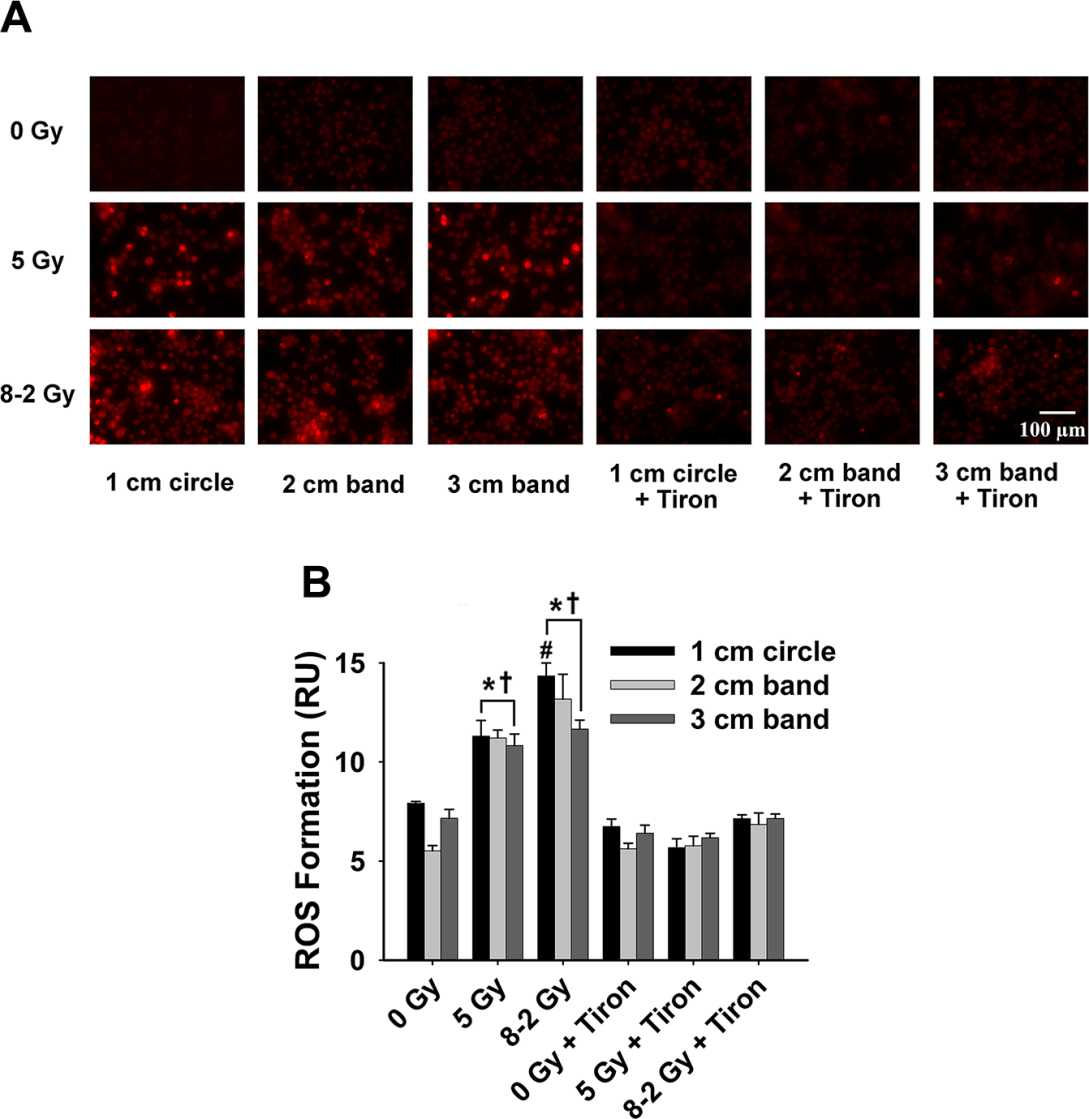
Intracellular ROS formation at 24 h after irradiation detected by DHE/ET fluorescence; data were measured from three randomly selected areas of 300–500 cells in 1 cm circle, 2 and 3 cm bands, respectively; 0 Gy ± Tiron *vs*. 5 Gy ± Tiron, and 8–2 Gy ± Tiron (**A**) Representative images of ROS formation (shown by red fluorescence); (**B**) Grouped ROS formation in 1 cm circle, 2 and 3 cm bands (mean ± SE); ^*^p < 0.05 *vs*. 0 Gy at the same circle/band; ^#^*p* < 0.05 *vs*. 5 Gy at the same circle/band; ^†^*p* < 0.05 *vs*. Tiron-treated group at the same circle/band and at the same dose.

**Figure 8:**
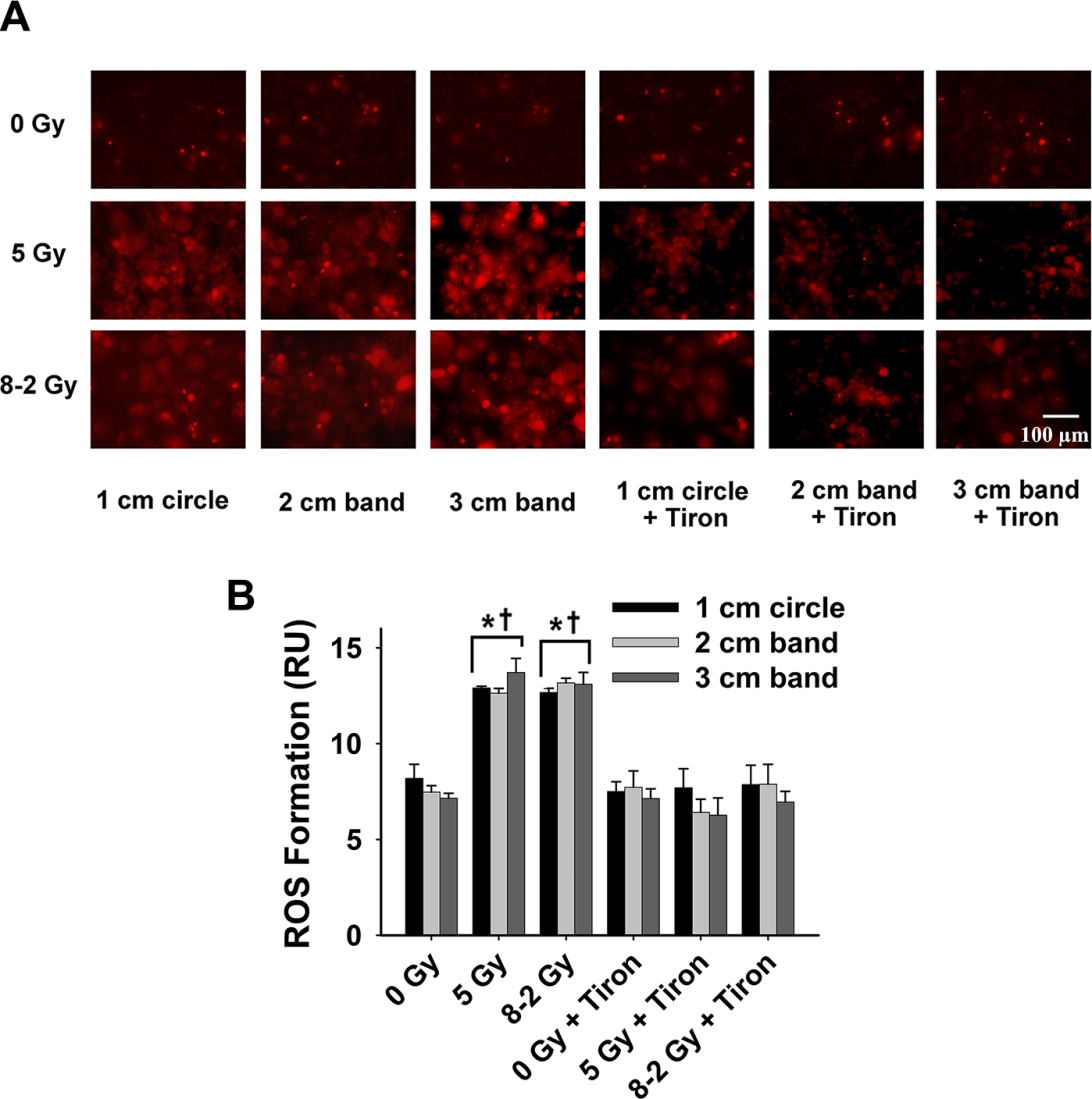
Intracellular ROS formation at 48 h after irradiation detected by DHE/ET fluorescence; data were measured from three randomly selected areas of 300–500 cells in 1 cm circle, 2 and 3 cm bands, respectively; 0 Gy ± Tiron *vs*. 5 Gy ± Tiron, and 8–2 Gy ± Tiron (**A**) Representative images of ROS formation (shown by red fluorescence); (**B**) Grouped ROS formation in 1 cm circle, 2 and 3 cm bands (mean ± SE); ^*^p < 0.05 *vs*. 0 Gy at the same circle/band; ^†^*p* < 0.05 *vs*. Tiron-treated group at the same circle/band and at the same dose.

We evaluated the intracellular ROS generation by comparing the levels of O_2_^·−^ formed at 2 h (Figure 6), 24 h (Figure 7) and 48 h (Figure 8) after GI (8–2 Gy) and UI (5 Gy). Both irradiation treatment groups had significantly elevated O_2_^·−^ levels, which were scavenged by Tiron application. The O_2_^·−^ generation at 2 h after irradiation was represented by ET fluorescence in Figure 6A. Quantified ROS fluorescence was summarized in Figure 6B. For UI of 5 Gy treated group, there was no significant difference in O_2_^·−^ formation across the 3 bands (*p* = 0.337). However, under GI (8–2 Gy), a marked reduction of O_2_^·−^ formation was observed from regions of 1 cm circle to 3 cm band (Figure 6B, *p* < 0.005 for 1 cm *vs*. 3 cm and *p* < 0.05 for 2 cm *vs*. 3 cm). The volume-average doses that were delivered to regions of 1 cm circle, 2 cm, and 3 cm bands were calculated as 7.3 Gy, 4.8 Gy, and 2.4 Gy, respectively. Notably, ROS production at the 2 cm band was significantly higher (*p* < 0.05) in GI than UI cells (Figure 6B) despite lower volume-average dose (4.8 Gy in GI *vs*. 5 Gy in UI).

At 24 h, UI induced no significant difference in O_2_^·−^ formation across the three bands (*p* = 0.847) (Figure 7). ROS production reached a similar level in 1 cm, 2 cm and 3 cm band, although different dosages were delivered across the bands in GI (*p* = 0.167, Figure 7B). ROS formation at 48 h after irradiation was summarized in Figure 8. No significant differences in ROS production were detected across the three bands (1 cm, 2 cm and 3 cm) in the UI group (*p* = 0.292) or in the GI group (*p* = 0.651). Comparing the two irradiation groups (UI *vs*. GI), no significant differences in ROS were observed from the comparisons between the pairs of 1 cm, 2 cm, and 3 cm bands, respectively.

## DISCUSSION

Our study suggests that GI is superior to UI in both redox advantage and toxic dosage to surrounding tissues. ROS play a role in many pathophysiological responses promoting cancer and senescence [22, 23]. Specifically, we have demonstrated that extracellular ROS were significantly increased following irradiation in both GI and UI (Figure 1A). SOD-treated groups attenuated the IR-induced ROS generation which provided strong evidence that O_2_^·−^ is the major type of ROS that was transmitted from irradiated cells to extracellular media. Previous research has shown that extracellular O_2_^·−^ could directly mediate intercellular bystander effects [24, 25]. Low-dose gamma irradiation significantly boosted extracellular O_2_^·−^ generation in tumor cells as a response of bystander effects, which was triggered possibly by tumor growth factor-beta1(TGF-β1) [26]. O_2_^·−^ and other relatively stable ROS, such as hydrogen peroxide (H_2_O_2_) have been implicated in activation signaling pathways in neighboring cells by regulating stress-related proteins such as MAPK and p53 under low doses of irradiation exposure [27, 28]. The mechanism for the increase of extracellular O_2_^·−^ is complex and could be associated with an enhanced inflammatory response such as TNF-α overexpression [29]. Other ROS which are derived from O_2_^·−^, such as H_2_O_2_, may cause damage towards the normal or less-irradiated cells by giving sufficient perfusion time, exacerbating bystander effects [18, 27, 30]. Therefore, these observations suggest a potential mechanism underlying bystander effects by spreading “damage” to neighboring cells via an extracellular ROS-dependent pathway.

From cell viability data as shown in Figure 1B, Figure 2 and Figure 3, cell survivals were reduced in GI groups compared to control and UI groups at 24 h, suggesting a more damaging effect induced by GI at 24 h. Interestingly, the survival rate detected using Trypan Blue partially rebounded at 48 h, possibly due to the cellular proliferation or repair mechanism of cancer cells (Figure 2B). The trend of cell viability observed over the time is consistent with our extracellular ROS formation, which markedly increased from 2 h to 24 h following GI (Figure 1A). ROS generation has also been implicated in mediating cellular proliferation and apoptosis [31, 32]. In fact, studies involving certain cell lines have proposed apoptosis pathways as a potential mechanism underlying bystander effects-related cell death [33]. Accordingly, our cell viability data (Figure 3) were highly consistent with the apoptosis analysis (Figure 5), indicating irradiation-induced apoptosis may affect cancer cell viability following irradiation. Although both UI (5 Gy) and GI (8–2 Gy) groups showed an enhanced cell apoptosis and declining cell survival at 24 h, the more cellular damaging effects (declined viability and enhanced apoptosis) were observed in the GI (8–2 Gy) group (Figure 3 and 5). The irradiation dosage levels at the 2 and 3 cm bands of GI (8–2 Gy) were similar (~2 cm) or even lower (~3 cm) than the corresponding regions of UI (5 Gy). However, the cancer-cell killing effects of GI (8–2 Gy) were more significant than that in UI (5 Gy) at all three regions at 24 h and 48 h. These observations may be due to the bystander effects in the 2 and 3 cm bands of GI (8–2 Gy), mediated by triggering the cellular apoptosis pathways in the lower-dosage regions. Thus, ROS, one of the key signaling molecules involved in apoptosis initiation, may likely play an important role in the current bystander phenomenon [34]. GI caused lower cell viability, likely regulated by an extracellular ROS-involved bystander mechanism. The estimation of ED, based on conventional linear-quadratic model, illustrated the advantages of GI over UI. In Figure 1C, GI (8–2 Gy from center to edge) showed a higher ED than UI (5 Gy) at 2 h and 24 h, while its volume-average dose of 3.7 Gy (proportional to integral dose) was lower than that of UI (5 Gy). This suggests a stronger radiation efficacy in GI than that in UI via potential bystander effects.

Furthermore, irradiation stress is known to boost ROS production, acting as a secondary messenger by propagating pro-inflammatory signals or causing oxidative damage [35–37]. To examine the redox scheme and the potential role of bystander effects, the intracellular ROS formation, regarded as an index of oxidative stress, was studied in different gradient irradiated regions. As shown in Figure 6, we found that higher doses resulted in larger ROS generation across the 3 bands in GI (8–2 Gy) at 2 h after irradiation. Particularly, ROS formation at 2 cm bands of GI (volume-average dose 4.8 Gy) is ~11% higher than UI (5 Gy) at 2 h, implying the involvement of a radiation-induced bystander effects (Figure 6B). Our hypothesis is that oxidative stress manifested in the area under higher irradiation dose “migrated” to the region of lower irradiation dose, likely via bystander effects. This hypothesis can be supported by the observation that there were no significant differences in intracellular ROS levels between the 3 bands in GI at 48 h after irradiation (Figure 8). That being said, ROS formation eventually displayed a more homogeneous pattern compared to differentiated levels of ROS distribution observed previously at 2 h. Although the free radicals triggered by irradiation are regarded as short-lived (10^−5^s) and slow-diffused [6], the relatively stable downstream mediators could further this bystander effects.

**Figure 9:**
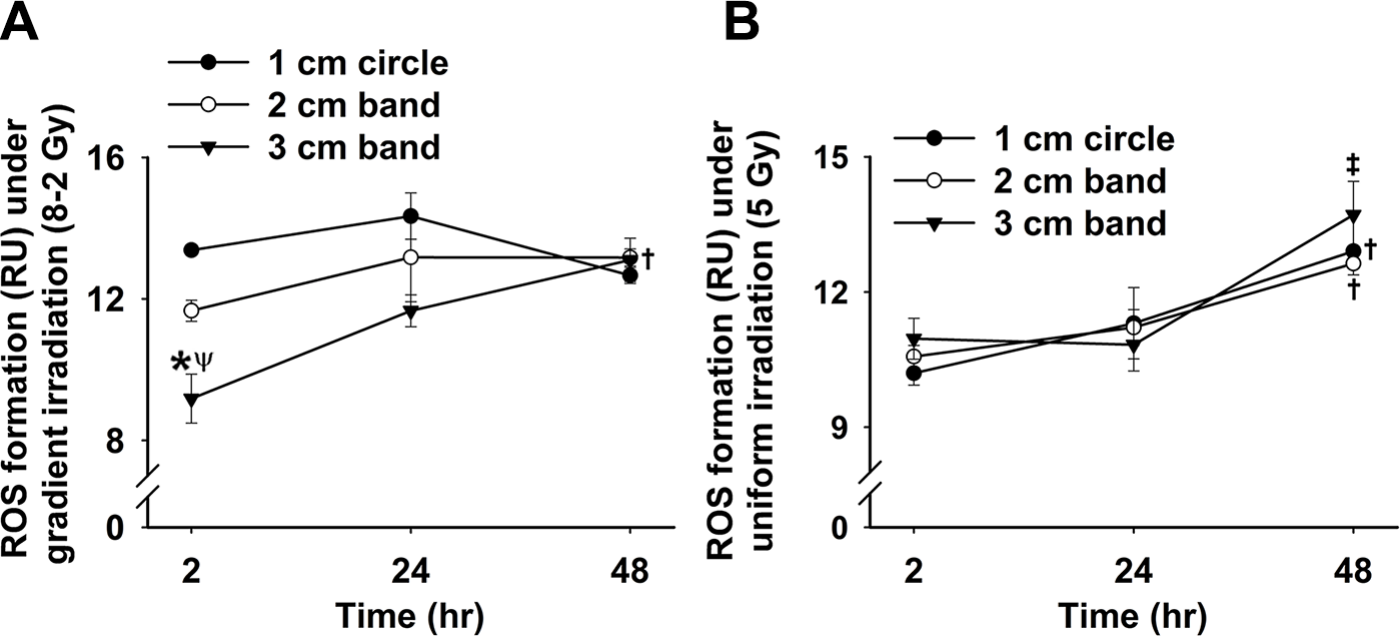
Grouped data showing intracellular ROS formation at 2, 24 and 48 h after irradiation under GI (**A**) and UI (**B**) across the three regions (measured from three randomly selected areas of 300–500 cells in 1 cm circle, 2 and 3 cm bands, respectively; mean ± SE). *p < 0.05 *vs*.1 cm circle at the same time; ^ψ^*p* < 0.05 *vs*. 2 cm band at the same time; ^†^*p* < 0.05 vs. 2 h point at the same circle/band; ^‡^*p* < 0.05 *vs*. 24 h point at the same circle/band.

Our result suggests a potential advantage of GI therapy over UI in reducing the damage to neighboring healthy cells. In the current experiment, the outer circular band of GI receives a dosage of only ~2 Gy as compared to 5 Gy in the outer band of UI region. Accordingly, GI would cause less damage to the neighboring cells due to lower marginal dosage profiles. Additionally, antioxidant Tiron treatment completely diminished ET fluorescence, thereby confirming that the signals are derived from the presence of intracellular ROS [38]. We believe that areas of lower irradiation dosages could be subjected to more profound bystander effects. For example, the 3 cm band in GI that received the lowest dose (volume-average dose 2.4 Gy) achieved the most significant intracellular ROS increase after 48 h (Figure 9A). However, no marked alteration of intracellular ROS levels can be detected in the 1 cm band of GI (volume-average dose 7.3 Gy) (Figure 9A). This finding could be explained by the previous study which showed that lower-dose radiations induce stronger bystander effects, attributing to the saturation effect at high-dose irradiations [10]. Widel et al. demonstrated that ROS generation induced by UV radiation was higher in cells influenced by bystander effects than those directly UV-exposed cells [39]. Furthermore, extracellular ROS response to irradiation reaches peak levels at 24 h after irradiation in both GI and UI (Figure 1A); whereas interestingly, intracellular ROS display completely different patterns between GI (Figure 9A) and UI (Figure 9B) groups. ROS (superoxide) are mostly impermeable to cell membranes due to its polarity [40, 41]. Further evidence has shown that the intracellular ROS migration could be independent of anion channels on the membrane [42]. Thus, it is speculated that intra- and extracellular ROS could be generated from different sources [38]. Accordingly, it is not surprising to observe that the intra- and extracellular ROS showed different production patterns over time, likely playing a coordinated role in mediating bystander effects in our study. These results also suggest the potential complex mechanisms underlying the intra- and extracellular ROS production due to GI and UI, are yet to be determined.

While it is essential to develop strategies to maximize the damaging effect in tumor cells and minimize the damaging effect in normal cells, understanding biological molecular mechanisms of bystander effects induced by radiation (e.g., cytokine signaling, redox regulation) can provide valuable insights into existing and future cancer radiotherapy. Nikjoo et al. in 2003 constructed a model to quantify the bystander effects induced by radiation [43]. They concluded that the distribution of bystander effects via signaling molecules in areas of lower dose radiation requires certain conditions such as the generation of molecules and sufficient diffusion time. This could explain the delayed ROS increase in human breast cancer cells with GI, as significant extracellular ROS elevation was only observed at 24 h following GI in our study. Interestingly, an increased extracellular ROS formation was also observed in UI (5 Gy)-irradiated cells (Figure 1A). This suggests that bystander effects may also peak in the UI group at 24 h, although the effects may not be as strong as that in the GI-irradiated cells. Moreover, Li et al. in 2013 reported that the increase in ROS production in hematoma cells induced by alpha-particles might trigger bystander effects which are regulated by a p53-depdendent pathway [44]. Butterworth et al. explored the clinical relevance of radiation-induced bystander effects and proposed the utilization of biologically optimized *in vivo* radiotherapy to improve the specificity of the treatment [4]. One of the challenges in these studies is how to determine the characteristics of irradiation such as radiation type, quality, and dose for high efficacy of cancer therapy. Accordingly, it is imperative to investigate the ideal dosage for maximal bystander effects and elucidates the exact molecular mechanism of GI.

There are limitations to *in vitro* studies in radiation-induced bystander effects due to the lack of consideration of the complex interactions in biological systems. *Ex vivo* and *in vivo* studies on GI are recommended in order to fully exploit and validate its clinical relevance to cancer radiotherapy. Since ROS play an essential role in irradiation-induced bystander response, cancer radiotherapy could be improved by developing more effective antioxidant treatment in addition to IR therapy. Accordingly, monitoring the activity level of SOD under irradiation may be a particularly promising approach [45]. In conclusion, our study indicates a better therapeutic effect of GI (8–2 Gy) compared to UI (5 Gy) regarding lower cell viability and higher apoptosis. Higher levels of extracellular ROS production in GI may play a role in spreading bystander signals from high to low dose irradiation regions and mediating cell apoptosis. We suggest a potential advantage of GI over UI in both enhancing ED to the target cells and mitigating the damaging effect to neighboring healthy cells.

## MATERIALS AND METHODS

### Cell culture and irradiation experiments

**Figure 10:**
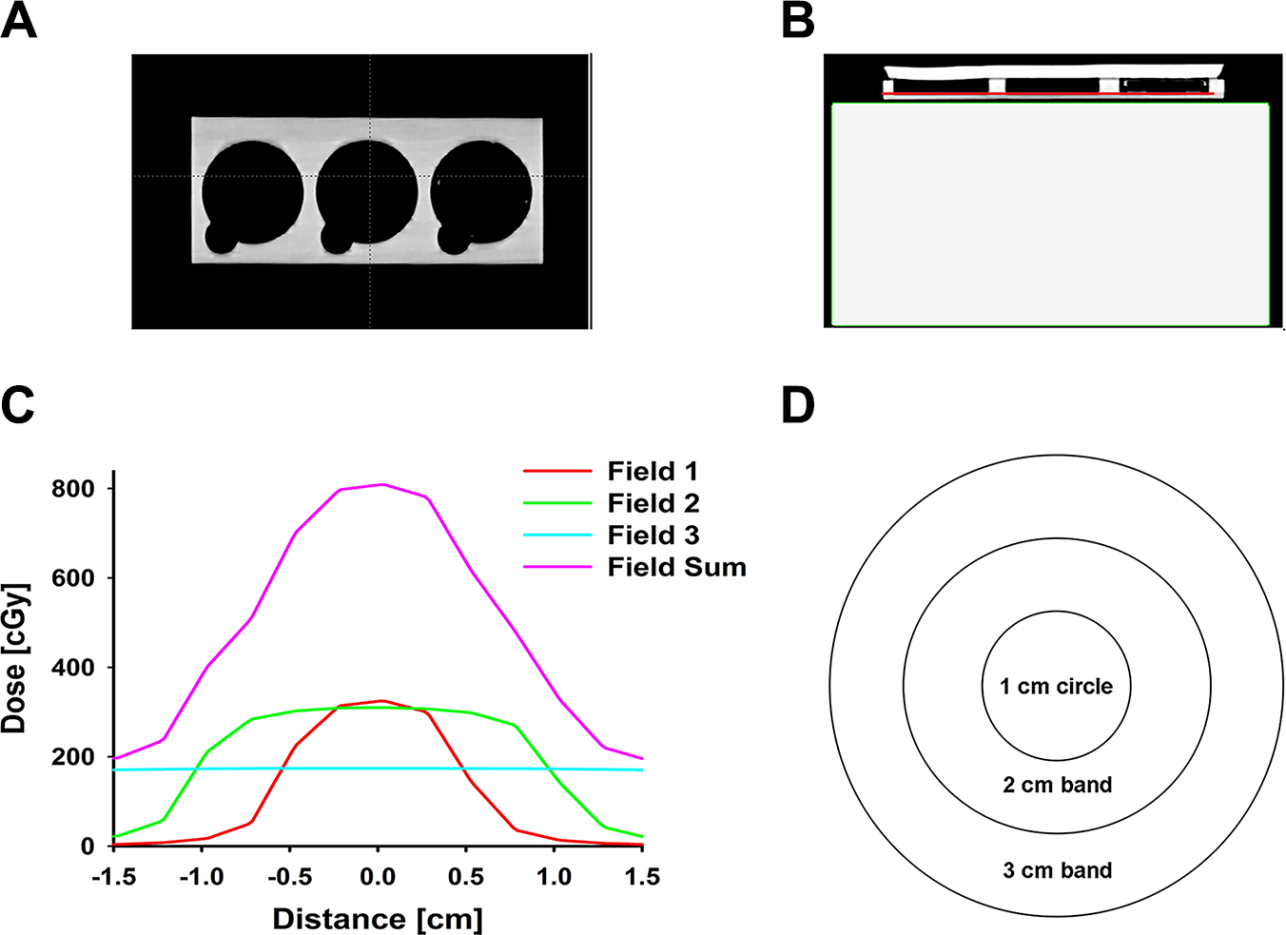
Irradiation design (**A**) Coronal slices of acrylic blocks with three cell plate holders; (**B**) Axial slices of acrylic blocks and solid water slabs (the red line indicates where the dose profiles to be taken); (**C**) Typical dose profiles of field 1 (orange), 2 (green), 3 (cyan), and the summation of the three fields (purple). Each plate was irradiated by three fields: Field 1, field size 1 cm × 1 cm, 500 MU, dose rate 600 cGy/min, energy 6 MV; Field 2, field size 2 cm × 2 cm, 400 MU, dose rate 600 cGy/min, energy 6 MV; Field 3, field size 5 cm × 5 cm, 200 MU, dose rate 600 cGy/min, energy 6 MV. Overlaps of the three fields generated a gradient dosage profile ranging from ~ 8 Gy at dish center to ~ 2 Gy at the edge of the irradiation dish; (**D**) A simplified diagram of three defined irradiation regions by their distances from the center of the plate: 1 cm circle, 2 cm circular band, and 3 cm circular band.

MCF-7 cell line was purchased from Cell Biolabs (San Diego, CA) and cultured on the BD Matrigel (Becton, Dickinson and Company (BD), Franklin Lakes, NJ, USA)-coated dish (3 cm diameter) two days before irradiation. Dulbecco's Modified Eagle Medium (DMEM, Life Technologies, Carlsbad, CA, USA) with 10% FBS (Life Technologies, Carlsbad, CA, USA) and 1% penicillin (Life Technologies, Carlsbad, CA, USA) were used as cell culture media. After cells uniformly adhered to the plate, irradiation was delivered. Cultured MCF-7 cell dishes were placed on the custom-made acrylic block to receive irradiation. These blocks were designed to contain three cell plate holders, as shown in Figure 10A. Solid water slabs were placed underneath the plate holder to provide backscatter, as shown in Figure 10B. The plate holder and solid water slabs were scanned together using a Siemens SOMATOM Sensation Open Syngo CT scanner (Siemens Medical Solutions, Mode #: 49445, Erlangen, Germany). Varian Eclipse treatment planning system (Varian Medical Systems, version 10.0.42, Palo Alto, California, USA) was used to design the irradiation profile, calculated at the red line location as shown in Figure 10B.

Under GI, each plate was irradiated by three fields (Figure 10C): Field 1, field size 1 cm × 1 cm, 500 MU, dose rate 600 cGy/min, energy 6 MV; Field 2, field size 2 cm × 2 cm, 400 MU, dose rate 600 cGy/min, energy 6 MV; Field 3, field size 5 cm × 5 cm, 200 MU, dose rate 600 cGy/min, energy 6 MV. Overlaps of these three fields (the purple line in Figure 10C) generated a gradient dosage profile ranging from ~ 8 Gy at dish center decreasing to ~ 2 Gy at the edge of the dish (1.5 cm radius). Three typical irradiation bands were defined based on their distance from the center of the culture dish (Figure 10D): i) Central 0.5 cm-radius circles were defined as 1 cm circles; ii) Circular bands between 0.5 cm and 1.0 cm were defined as the 2 cm bands; iii) Circular bands between 1.0 cm and 1.5 cm were defined as the 3 cm bands. The estimated volume-average dose for the three bands, using normalized integral dose in the corresponding band range, were 7.3 Gy, 4.8 Gy, and 2.4 Gy for 1 cm, 2 cm, and 3 cm bands, respectively. The estimated volume-average dose for the overall gradient field area (8–2 Gy) was 3.7 Gy.

For UI, the entire phantom was irradiated with a 30 cm × 30 cm field size for a total of 500 MU at a 600 MU/min dose rate with energy 6 MV. The 30 cm size is large enough to cover the entire phantom. Each plate was irradiated for a uniform dose of 5.1 Gy. The control cell dish group followed the same protocol without receiving any irradiation.

### Extracellular ROS detection after irradiation and cell number counting

The extracellular ROS formation was monitored by measuring the cytochrome *c* (Sigma-Aldrich Corporation, St. Louis, MO, USA) reduction at 2, 24 and 48 h intervals following irradiation using Nanodrop 2000 spectrophotometer (Thermal Scientific, MA, USA) [29]. Cells were incubated with 5 μM cytochrome *c* in cell culture medium [38]. Cytochrome *c*, when reduced by superoxide (O_2_^·−^, a major ROS), its absorbance at 550 nm is enhanced. The average absorbance at 540 nm and 560 nm was taken as the baseline and deducted from the absorbance peak at 550 nm to obtain the final reduction absorbance. This value is directly correlated to the concentration of extracellular O_2_^·–^ by an extinction coefficient of 18.5 × 10^3^ M^−1^cm^−1^ [46]. To determine the average extracellular O_2_^·−^ production per cell, cell number in each cytochrome *c*-treated group was counted at 2, 24 and 48 h after irradiation using Cellometer Mini (Nexcelom Bioscience, MA, USA), respectively. To determine whether the detecting signals are caused by extracellular O_2_^·−^ in our models, SOD (1500 U/mL, Sigma-Aldrich Corporation, St. Louis, MO, USA), a membrane-impermeable scavenger of extracellular O_2_^·−^, were applied following previous protocols [29, 38].

### Cell viability assay using alamar blue

Cell viability was determined at 2, 24 and 48 h after irradiation using an Alamar Blue assay kit (Life Technologies, Carlsbad, CA, USA) [47, 48]. 10% (v/v) of Alamar blue solution was kept in each dish with medium and the cells were further incubated at 37°C for 4 hours. After incubation, the cell culture medium was transferred to the 96-well plate for fluorescence analysis by microplate reader with excitation wavelength at 550 nm and emission wavelength at 590 nm (Spectra Max M2, Molecular Devices Corporation, Sunnyvale, CA) [49].

### Cell viability assay using trypan blue

To confirm the cell viability results from Alamar Blue, the current study also used Trypan Blue staining (Life Technologies, Carlsbad, CA, USA) to quantify the average cell viability of each dish at 2, 24, and 48 h after irradiation [50]. Cells were detached using trypsin (Life Technologies, Carlsbad, CA, USA) and re-suspended in DMEM. A 50 uL sample of cells was mixed with 0.4% Trypan Blue solution by 1:1. The cell viability was assessed and calculated using Cellometer Mini (Nexcelom Bioscience, Lawrence, MA, USA).

### Region-specific cell viability assay using propidium iodide (PI)

PI staining (Sigma-Aldrich Corporation, St. Louis, MO, USA) was used to examine the cell viability in the 1 cm circle, as well as the 2 and 3 cm bands of the irradiation area, providing more spacial information of the cell response to our designed irradiation intensities [34]. At 2, 24, and 48 h after irradiation, cancer cells were incubated with 7.5 μM PI buffer for 15 min at 37°C and then washed twice with PBS. PI fluorescence was monitored via Nikon Eclipse TS 100 microscope (Nikon Corporation, Tokyo, Japan). The setup for fluorescence imaging of PI fluorescence was as follows: Xenon lamp power (LPS-100, Photon Technology International, Inc. (PTI), Birmingham, NJ, USA); DeltaRAM X High-Speed Multi-Wavelength Illuminator LPS-100 (Photon Technology International, Inc. (PTI), Birmingham, NJ, USA); Nikon Eclipse TS 100 microscope and CCD camera (Nikon Corporation, Tokyo, Japan); ET excitation, 535 ± 15 nm; ET emission, 617 ± 37.5 nm, objective × 20. The emitted signal was captured and presented as an image of 1392 × 1040 pixels on a computer monitor using Macro-ImageJ software (National Institutes of Health, Bethesda, MD, USA). Three random areas were selected from each band for the cell viability assay. The mean fluorescence was analyzed using Adobe Photoshop CS6 (64 Bit) software (Adobe Systems Inc., San Jose, CA, USA) to determine the cell viability.

### Cell apoptosis assay using annexin V-Cy3

Annexin V-Cy3 (Abcam, Cambridge, United Kingdom) was employed to monitor cellular apoptosis levels after irradiation [51]. Cancer cells were loaded with ~30 ug/ml Annexin V-Cy3 in a binding buffer (Abcam, Cambridge, United Kingdom) for 5 min in the dark at 2, 24, and 48 h after irradiation. Annexin V-Cy3 fluorescence was detected via Nikon Eclipse TS 100 microscope (Nikon Corporation, Tokyo, Japan). The imaging setup was as follows: ET excitation, 543 ± 15 nm; ET emission, 610 ± 37.5; objective × 20. The emitted signal was captured and recorded as an image of 1392 × 1040 pixels on a computer monitor using Macro-ImageJ software (National Institutes of Health, Bethesda, MD, USA). Three random areas were selected from each band for apoptosis evaluation of the band. The mean fluorescence intensity of each area was analyzed using Adobe Photoshop CS6 (64 Bit) software (Adobe Systems Inc., San Jose, CA, USA) to determine cell apoptosis levels.

### Intracellular ROS detection after irradiation

The intracellular ROS production was monitored at 2, 24 and 48 h after irradiation. Cancer cells were loaded with 5 μM dihydroethidium (DHE)/ethidium (ET) (Life Technologies, Carlsbad, CA, USA), a fluorescence probe primarily targeted for O_2_^·−^, for 30 min at 37°C and then washed out with PBS (Life Technologies, Carlsbad, CA, USA). The setup for fluorescence imaging of ROS was as follows: ET excitation, 543 ± 15 nm; ET emission, 610 ± 37.5 nm, objective × 20. The emitted signal was captured and presented as an image of 1392 × 1040 pixels on a computer monitor using Macro-ImageJ software (National Institutes of Health, Bethesda, MD, USA). Three random areas were selected from each typical band as a representative profile of ROS generation of that band. The mean fluorescence intensity of each area was analyzed using Adobe Photoshop CS6 (64 Bit) software (Adobe Systems Inc., San Jose, CA, USA). In order to determine the specificity of O_2_^·−^ probes (ET), we applied Tiron (0.25 mM, an intracellular scavenger of O_2_^·−^, Sigma-Aldrich Corporation, St. Louis, MO, USA) into the loading solution. These treatments would determine whether the detecting signals were caused by intracellular ROS or other chemicals released from our cell models [38].

### Statistical analysis

Results are expressed as mean ± SE (JMP, SAS Institute, NC). Data were analyzed using one-way ANOVA with time, dosage and irradiation location. Statistical difference between various treatment groups were interpreted and displayed via Bonferroni post-hoc test using JMP (SAS Institute, Cary, NC). *p* < 0.05 was regarded as statistically different.
